# A descriptive survey on the use of resuscitative endovascular balloon occlusion of the aorta (REBOA) for pelvic fractures at US level I trauma centers

**DOI:** 10.1186/s13037-019-0223-3

**Published:** 2019-12-13

**Authors:** Stephanie Jarvis, Michael Kelly, Charles Mains, Chad Corrigan, Nimesh Patel, Matthew Carrick, Mark Lieser, Kaysie Banton, David Bar-Or

**Affiliations:** 1 Trauma Research, LLC. 383 Corona St. #319, Denver, CO 80218 USA; 2 Orthopedic Trauma Surgeon, Penrose Hospital, 2222 North Nevada Ave. Colorado Springs, Englewood, CO 80907 USA; 3 Trauma Systems Director, Centura Health Systems, 9100 E. Mineral Circle, Centennial, CO 80112 USA; 4 Orthopedic Trauma Surgeon, Wesley Medical Center, 550 N Hillside St., Wichita, KS 67214 USA; 5 Orthopedic Trauma Surgeon, St. Anthony Hospital, 11600 West 2nd Place, Lakewood, CO 80228 USA; 6 Trauma Medical Director, Medical City Plano, 3901 West 15th Street, Plano, TX 75075 USA; 7 Trauma Medical Director, Research Medical Center, 2316 East Meyer Blvd, Kansas City, MO 64132 USA; 8 Trauma Medical Director, Swedish Medical Center, 501 E. Hampdem Ave., Englewood, CO 80113 USA

**Keywords:** Pelvic fracture management, Resuscitative endovascular balloon occlusion of the aorta (REBOA), Level I trauma center

## Abstract

**Background:**

Resuscitative endovascular balloon occlusion of the aorta (REBOA) is not widely adopted for pelvic fracture management. Western Trauma Association recommends REBOA for hemodynamically unstable pelvic fractures, whereas Eastern Association for the Surgery of Trauma and Advanced Trauma Life Support do not.

**Method:**

Utilizing a prospective cross-sectional survey, all 158 trauma medical directors at American College of Surgeons-verified Level I trauma centers were emailed survey invitations. The study aimed to determine the rate of REBOA use, REBOA indicators, and the treatment sequence of REBOA for hemodynamically unstable pelvic fractures.

**Results:**

Of those invited, 25% (40/158) participated and 90% (36/40) completed the survey. Nearly half of trauma centers [42% (15/36)] use REBOA for pelvic fracture management. All participants included hemodynamic instability as an indicator for REBOA placement in pelvic fractures. In addition to hemodynamic instability, 29% (4/14) stated REBOA is used for patients who are ineligible for angioembolization, 14% (2/14) use REBOA when interventional radiology is unavailable, 7% (1/14) use REBOA for patients with a negative FAST. Fifty percent (7/14) responded that hemodynamically unstable pelvic fractures exclusively indicates REBOA placement. Hemodynamic instability for pelvic fractures was most commonly defined as systolic blood pressure of < 90 [56% (20/36)]. At centers using REBOA, REBOA was the first line of treatment for hemodynamically unstable pelvic fractures 40% (6/15) of the time.

**Conclusions:**

There is little consensus on REBOA use for pelvic fractures at US Level I Trauma Centers, except that hemodynamically unstable pelvic fractures consistently indicated REBOA use.

## Background

Resuscitative endovascular balloon occlusion of the aorta (REBOA) for temporary hemorrhage control is an alternative to aortic cross-clamping that has been used for patients with hemodynamically unstable pelvic fractures; however its use is not widely adopted [1–3]. A REBOA can be temporarily placed until definitive hemorrhage control [1, 2]. For pelvic fracture hemorrhage, REBOA may perform best if deployed in zone III, from the lowest renal artery to the aortic bifurcation, but can also be deployed in zone I, the supra-celiac or descending aorta [1, 4, 5]. Both the effectiveness and safety of REBOA for pelvic fracture management are not well established as data from current ongoing prospective studies comparing REBOA to other hemorrhage control methods are not yet available [6]. However, the American College of Surgeons (ACS) has outlined potential complications of REBOA including: arterial disruption, dissection, pseudoaneurysm, hematoma, thromboembolism, extremity ischemia, balloon rupture, spinal cord injuries due to prolonged organ ischemia, and suggest that acute care surgeons or an interventionalist trained in REBOA should perform the intervention [7].

Recommendations regarding REBOA use for pelvic fracture management vary across published guidelines. The Eastern Association for the Surgery of Trauma (EAST) and Advanced Trauma Life Support (ATLS) guidelines do not include the utilization of REBOA for pelvic fracture management [8, 9]. The Western Trauma Association (WTA) recommends to use the following treatment approaches after a negative focused assessment with sonography in trauma (FAST) or diagnostic peritoneal aspiration (DPA): pelvic stabilization, preperitoneal pelvic packing, and REBOA, which they state are complementary and not mutually exclusive that can be performed immediately [10]. The Trauma Quality Improvement Project (TQIP) recommends REBOA for patients in extremis solely from pelvic bleeding as a potential alternative initial intervention or in addition to pelvic stabilization with preperitoneal pelvic packing [11]. The World Society of Emergency Surgeons (WSES) provides a more definitive guideline, stating that REBOA may provide an alternative method to aortic cross-clamping for severe, hemodynamically unstable pelvic fractures [4]. The WSES guideline lists REBOA along with temporary mechanical stabilization and angioembolization as treatment options with the application of preperitoneal pelvic packing as the first line of treatment for severe hemodynamically unstable pelvic fractures [4]. Additionally, WSES recommends that partial or intermittent REBOA be considered to decrease occlusion time and ischemic insult [4]. Intermittent balloon inflation and deflation or partial deflation of REBOA can help restore hemodynamic stability but has not been well practiced [1, 7].

Evident by varying recommendations in published guidelines, it remains uncertain whether REBOA is effective for hemodynamically unstable patients with pelvic fractures. In fact, Stahel et al. state that quality scientific data from prospective trials and validation studies are needed to determine the value of REBOA for the management of hemorrhagic shock from bleeding pelvic fractures. Additionally, Constantini et al. stated that there is currently no consensus on the optimal management of pelvic fractures [1]. Because of this, it is important to know how often REBOA is used in Level I trauma centers for pelvic fracture management across the United States (US). The purpose of this study was to determine the rate of REBOA use at Level I trauma centers, in what scenario REBOA is used, the sequence of REBOA use for a hemodynamically unstable pelvic fractures, and factors associated with REBOA use.

## Methods

This prospective cross-sectional survey of trauma medical directors at US Level I trauma centers was approved by the Western Institutional Review Board. Survey questions were generated by the coauthors and the study was tested by two trauma medical directors. The survey was conducted on Survey Monkey Inc. (San Mateo, California; www.surveymonkey.com) and included 46 questions; questions with responses pertinent to this paper are found in the manuscript tables. Any question could be skipped for any reason and questions deemed irrelevant were skipped based on responses to previous closed-ended questions using SurveyMonkey’s “skip logic”. For example, participants who indicated that REBOA was not used at their trauma center were not asked what indicates the use of REBOA.

All 158 trauma medical directors at American College of Surgeons (ACS) verified Level I trauma centers were invited to participate via email. ACS-verified Level I trauma centers were identified from the ACS website; to view a list of those invited to participate please view Appendix 1. The list of ACS-verified Level I trauma centers was compiled prior to sending the initial survey invitation notice in February 2018 and was updated in April 2018 with additional centers. Contact information was obtained from individual Level I trauma center’s websites or via telephone call to the Level I trauma centers. Six email invitations containing the approved consent form with a partial waiver of consent were sent every two weeks from 3/1/2018 to 6/26/2018. Before the 5th and 6th email reminder, trauma medical directors were called to verify they received the email invitation if they had not already responded. The trauma medical director or an assigned college were allowed to take the survey and will be referred to as “participants”.

Hospitals were dichotomized into two groups: those who used REBOA and those who did not use REBOA for pelvic fracture management. Responses were compared between the two groups to see if they varied by: having a guideline in place for pelvic fracture management, the year the guideline was implemented, what guideline was followed, how long the trauma center has been ACS-verified as Level I, and the volume of trauma admissions in 2017. The volume of trauma admissions was categorized as high volume, more than 1500 patients admitted in 2017, and low volume, less than or equal to 1500 patients admitted in 2017. Categorical and dichotomous questions were summarized as counts and proportions. The denominator in each proportion presented represents the number of participants who answered the question; because participants were allowed to skip questions and SurveyMonkey’s skip logic was used, the denominator varies for each individual question. Responses were compared using Fisher’s exact test or chi-squared when appropriate. An alpha of 0.05 was used for significance.

## Results

Of the 158 Level I trauma centers invited to participate, 25% (40/158) responded to the survey and 90% (36/40) completed the survey. Of the participating Level I trauma centers, 73% (29/40) had a treatment guideline in place for pelvic fractures; guidelines were implemented as early as 2005, with most participants [37% (7/19)] implementing their guideline in 2016, Table 1. A majority [79% (23/29)] of trauma centers’ guidelines for pelvic fractures were based on a published guideline, and EAST was the most common guideline followed [43% (9/21)]. A majority [58% (23/40)] of participants indicated their hospital has been a Level I trauma center for more than 10 years and a majority of participants indicated that their hospital had a high volume (> 1500) of trauma admissions in 2017 [90% (36/40)].

**Table 1:** Characteristics of Participating Level I Trauma Centers and Their Pelvic Fracture Management Guidelines

Survey Question	Responses	% (n)	n
Does your hospital have a guideline for pelvic fracture management?	Yes No	73% (29) 28% (11)	40
In what year was the current guideline for pelvic fracture management implemented? If unknown, please leave blank.	2005	5% (1)	19
	2006	5% (1)	
	2011	5% (1)	
	2013	16% (3)	
	2014	5% (1)	
	2015	21% (4)	
	2016	37% (7)	
	2017	5% (1)	
Is your hospital’s guideline for pelvic fracture management based on a published guideline?	Yes	79% (23)	29
	No	21% (6)	
What published guideline is your hospital following?	WTA	29% (6)	21
	EAST	43% (9)	
	TQIP	14% (3)	
	WSES	0	
	ATLS	10% (2)	
	Other (please specify): fill in the blank.^a^	5% (1)	
How long has your trauma center been an ACS-verified Level I trauma center?	≤ 1 year	5% (2)	40
	> 1 year to 2 years	15% (6)	
	> 2 years to 5 years	18% (7)	
	> 5 to 10 years	5% (2)	
	> 10 years	58% (23)	
How many trauma admissions did you have in 2017?	High volume (> 1500)	90% (36)	40
	Low volume (≤ 1500)	10% (4)	

*WTA* Western Trauma Association, *EAST* Eastern Trauma Association, *TQIP* Trauma Quality Improvement Project, *WSES* World Society of Emergency Surgeons, *ATLS* Advanced Trauma Life Support, ^a^ Participant indicated that protocol is based on the Orthopedic Trauma Association, *EAST*, *TQIP* and a literature review

Responses were split when asked if REBOA is used to treat hemodynamically unstable pelvic fractures, with 42% (15/36) responding yes and 58% (21/36) responding no (Table 2). There was a higher proportion of Level I trauma centers that used REBOA and had a guideline in place for pelvic fracture management, 87% (13/15), than among trauma centers that did not use REBOA for pelvic fracture management, 67% (14/21), though this difference observed was not statistically significant, *p* = 0.25 (Table 3). REBOA use was not significantly associated with the year that the hospital implemented the guideline for pelvic fracture management, *p* = 0.55; however, it appeared that hospitals using REBOA were following more recently published guidelines. REBOA use was not significantly associated with the guideline followed, *p* = 0.17. Half (5/10) of the centers that use REBOA were following the EAST guideline, whereas 36% (4/11) of the centers that did not use REBOA followed the EAST guideline. Thirty percent (3/10) of the centers that use REBOA followed TQIP’s guideline whereas none (0/11) of the centers that did not use REBOA followed the TQIP guideline. Although there was a higher proportion of hospitals using REBOA that were ACS-verified for more than ten years, 60% (9/15), than hospitals that did not use REBOA, 52% (11/21); REBOA use did not significantly vary based on the number of years that the hospital was ACS verified as a Level I trauma center, *p* = 0.94. REBOA was used more at high volume centers, 93% (14/15), than participants at low volume centers, 7% (1/15), however, this difference was also not statistically significant, *p* = 0.63.

**Table 2:** REBOA use at Participating Level I Trauma Centers

Survey Question	Responses	% (n)	n
Does your hospital use a REBOA to treat hemodynamically unstable pelvic fractures?	Yes	42% (15)	36
	No	58% (21)	
What indicates a patient with a pelvic fracture for REBOA?	HDU	50% (7)	14
	HDU & IR is unavailable	14% (2)	
	HDU & negative FAST	7% (1)	
	HDU & not a candidate for angioembolization	29% (4)	
In what order are the following treatments utilized for hemodynamically unstable pelvic fractures? REBOA, angioembolization, and pelvic packing	Angioembolization, PP, REBOA	33% (5/15)	15
	Angioembolization, REBOA, PP	13% (2/15)	
	PP, Angioembolization, REBOA	13% (2/15)	
	REBOA, Angioembolization, PP	20% (3/15)	
	REBOA, PP, Angioembolization	20% (3/15)	
Of those who said hemodynamic instability was the only indicator, what was the order of treatment? REBOA, angioembolization and pelvic packing	PP, Angioembolization, REBOA	33% (2/6)	6
	Angioembolization, PP, REBOA	33% (2/6)	
	Angioembolization, REBOA, PP	17% (1/6)	
	REBOA, Angioembolization, PP	17% (1/6)	

*REBOA* resuscitative endovascular balloon occlusion of the aorta, *HDU* hemodynamically unstable, *IR* interventional radiology, *FAST* focused assessment of sonography in trauma, *PP* pelvic packing.

**Table 3:** Examining Associations with REBOA Use

	Does Not Use REBOA *n* = 21	Uses REBOA *n* = 15	n	p
Does your hospital have a guideline for the management of pelvic fractures?				
No	33% (7)	13% (2)	36	0.25
Yes	67% (14)	87% (13)		
In what year was your guideline for the management of pelvic fractures implemented?				
2005	11% (1)	0	18	0.55
2006	11% (1)	0		
2011	0	11% (1)		
2013	11% (1)	22% (2)		
2014	11% (1)	0		
2015	22% (2)	11% (1)		
2016	22% (2)	56% (5)		
2017	11% (1)	0		
What published guideline does your hospital follow?				
ATLS	18% (2)	0	21	0.17
EAST	36% (4)	50% (5)		
TQIP	0	30% (3)		
WTA	36% (4)	20% (2)		
Other^a^	9% (1)	0		
How long has your trauma center been a Level I trauma center?				
≤ 1 year	5% (1)	7% (1)	36	0.94
> 1 year to 2 years	19% (4)	7% (1)		
> 2 years to 5 years	19% (4)	20% (3)		
> 5 to 10 years	5% (1)	7% (1)		
> 10 years	52% (11)	60% (9)		
How many trauma admissions did your site have in 2017?				
Low volume (≤ 1500)	14% (3)	7% (1)	36	0.63
High volume (> 1500)	86% (18)	93% (14)		

*ATLS* Advanced Trauma Life Support, *EAST* Eastern Association for The Surgery of Trauma, *TQIP* Trauma Quality Improvement Project, *WTA* Western Trauma Association. ^a^ Participant indicated that protocol is based on the Orthopedic Trauma Association, *EAST*, *TQIP* and a literature review

### Hospitals using REBOA for pelvic fracture management

Among hospitals that use REBOA, more hospitals had a pelvic fracture management guideline in place [87% (13/15)] than hospitals without a guideline in place [13% (2/15)]. A majority of participants had guidelines implemented in 2016, [56% (5/9)] and half (5/10) were following the EAST guideline. Sixty percent (9/15) of the trauma centers had been ACS-verified Level I hospitals for more than ten years. Almost all [93% (14/15)] of the trauma centers had a high volume of trauma admissions in 2017. Given the choice of REBOA, pelvic packing, and angioembolization for treating hemodynamically unstable pelvic fractures, the most common sequence was angioembolization, pelvic packing and then REBOA [33% (5/15)] (Table 2). REBOA is used as the first line of treatment for hemodynamically unstable pelvic fractures for 40% (6/15) of the Level I trauma centers. Angioembolization was also used as the first line of treatment for hemodynamically unstable patients for 47% (7/15) of the Level I trauma centers and pelvic packing was used as the first line of treatment at 13% (2/15) of Level I trauma centers.

All participants included hemodynamic instability as an indicator for REBOA placement in pelvic fractures. In addition to hemodynamic instability, 29% (4/14) stated REBOA is used for patients who are ineligible for angioembolization, 14% (2/14) use REBOA when interventional radiology is unavailable, 7% (1/14) use REBOA for patients with a negative FAST. Fifty percent (7/14) responded that hemodynamically unstable pelvic fractures was used exclusively as an indicator for REBOA placement. Of the participants who indicated hemodynamic instability was the only indicator for REBOA in patients with pelvic fractures, only one center [17% (1/6)] used REBOA as the first line of treatment.

Participants were asked to indicate what measures are used to define hemodynamic instability: systolic blood pressure (SBP), heart rate (HR), lactate, international normalized ratio (INR), or “other: fill in the blank with response” (Table 4). All participants indicated that SBP is used to define hemodynamic instability for pelvic fractures. Seventy-five percent also use HR to define hemodynamic instability. The most common definitions of hemodynamic instability for pelvic fractures was SBP alone or SBP and HR. Other methods for defining hemodynamic instability included base deficit [29% (2/7)], rotational thromboelastography (ROTEM) or thromboelastography (TEG) [29% (2/7)], radiology [14% (1/7)], requiring transfusion [14% (1/7)], and diastasis at pubic symphysis [14% (1/7)]. The most common response cut-off for hypotension was SBP <  90 mmHg [56% (20/36)] but responses ranged from < 80 mmHg to ≤100 mmHg. An elevated heart rate was defined as > 100 bpm for 10 participants (37%) and >  120 bpm for another 10 (37%) participants. Of those who use lactate to define hemodynamic instability, the most common response was a lactate > 2 mg/dL [41% (7/17)]. Seven participants indicated that INR is used to define hemodynamic instability, most commonly defined by INR >  1.5.

**Table 4:** Defining Hemodynamic Instability for Patients with Pelvic Fractures

Survey Question	Responses	% (n)	n
What measures are used to define hemodynamic instability for pelvic fractures?	SBP	25% (9)	36
	SBP and HR	25% (9)	
	SBP, HR, and Lactate	14% (5)	
	SBP, HR, and Other	3% (1)	
	SBP, HR, Lactate, and INR	17% (6)	
	SBP, HR, Lactate, and Other	11% (4)	
	SBP, HR, Lactate, INR, and Other	6% (2)	
What measures are used to define a hemodynamic instability for pelvic fractures?	SBP	100% (36)	36
	HR	75% (27)	
	Lactate	47% (17)	
	INR	22% (8)	
	Other	19% (7)	
What “other” methods are used to define hemodynamic instability in pelvic fractures?	Base deficit	29% (2)	7
	ROTEM or TEG	29% (2)	
	Radiology	14% (1)	
	Requiring Transfusion	14% (1)	
	Diastasis at Pubic Symphysis	14% (1)	
How do you define measures of hemodynamic instability for pelvic fractures? Most common response (n)	SBP	< 90 (20)	36
	Heart Rate	> 100 (10), > 120 (10)	27
	Lactate	> 2 (7)	17
	INR	> 1.5 (3)	8
How do you define measures of hemodynamic instability for pelvic fractures? Range of responses.	SBP	< 80 - ≤ 100	27
	Heart Rate	< 60–125	36
	Lactate	> 2–10	17
	INR	> 1.1 - > 2.5	8
How are “other” measures of hemodynamic instability for pelvic fractures defined?	Base deficit	−5, 6	7
	ROTEM or TEG	Not defined	
	Radiology	Not defined	
	Requiring Transfusion Diastasis at Pubic Symphysis	Not defined > 2.5 cm	

*SBP* systolic blood pressure, *HR* heart rate, *INR* international normalized ratio, *ROTEM* rotational thromboelastography, *TEG* thromboelastography

## Discussion

This study was successful in determining the rate of REBOA use specifically for pelvic fracture management at Level I trauma centers, finding there is little consensus and less than half (42%) of the participating centers use REBOA for pelvic fracture management while the majority (58%) of hospitals do not use REBOA. The results of the survey also provide insight on the scenarios for which REBOA is used and the sequence of deployment for hemodynamically unstable pelvic fractures. REBOA use was not dependent on having a guideline for pelvic fracture management, how long the Level I trauma center had a guideline in place, what guideline the trauma center was following, how long the trauma center has been ACS-verified as Level I or how many trauma admissions the trauma center had in 2017.

Little data exits on the effectiveness of REBOA for pelvic fracture management; however previous studies have shown REBOA has been used for pelvic fractures. Benjamin et al. conducted an observational study in Europe on REBOA use from a databank of 72,677 adult trauma patients and found that 397 had a REBOA placed, 177 (45%) of those having hemorrhagic shock and a pelvic fracture [12]. However in this study they did not compare mortality rates across injury mechanisms and did not report complications specific to patients with pelvic fracture, so it is difficult to conclude on the success of REBOA use from their study [12]. Another retrospective study assessed patients previously admitted to determine if they were potentially amenable to REBOA, found only two patients with pelvic fractures without contraindications of 666 patients over two years; this small population of patients with hemodynamically unstable pelvic fractures eligible for REBOA may be part of the reason for the limited data on REBOA use for pelvic fractures [13]. In a recent American Association for the Surgery of Trauma (AAST) multicenter study of 11 Level I trauma centers, only one center used REBOA for pelvic fracture management, a far lower percentage of REBOA use for pelvic fracture hemorrhage than observed in this study [14].

It is not evident if using REBOA for pelvic fracture hemorrhage reduces mortality rates. Skitch et al. reported that studies in Japan have shown that REBOA has not improved mortality rates an can cause severe complications such as limb loss, but mention there are clinical differences in deployment between Japan and North American [3]. In 2017, Agri et al. stated that the efficiency of REBOA in terms of mortality and blood transfusions is unclear and Stahel et al. identified a need for more validation studies on REBOA for pelvic fracture management; nevertheless this study found that almost half of the participating centers were utilizing REBOA for pelvic fracture management [2, 15]. This could indicate a perception towards safety of REBOA despite a lack of evidence showing that specifically for pelvic fracture management [6, 7]. Martinelli et al. also conducted a retrospective review and found that 1% (13/2064) of patients with pelvic fractures were treated with REBOA [16]. They additionally found that non-survivors had a significantly longer time with REBOA deployed, however due to the small sample size of 13 patients, they could not adjust for the significantly higher injury severity score in the non-survivors [16]. In addition, they did not compare the mortality rates to similarly injured patients treated with alternative methods for hemorrhage control, so they cannot conclude if REBOA reduced mortality rates in patients with pelvic fractures [16].

In one case report that described using REBOA for a hemodynamically unstable pelvic fracture, the authors concluded that REBOA should only be used by experienced surgeons for pelvic fractures when they are able to rapidly start surgical rescue and suggest that some pelvic injuries may be contraindicated for REBOA [17]. However, contraindications for REBOA in patients with pelvic fractures remain unclear are not outlined in current treatment guidelines utilizing REBOA [4, 10, 11]. Although contraindications are not known, the results of this survey show that 40% of participants are utilizing REBOA for initial hemorrhage control, before pelvic packing or angioembolization. Biffl et al. described REBOA for pelvic fracture management and state that REBOA deployed in Zone III may prove to be the optimal means of immediate hemorrhage control [5]. They outline that future research should investigate if REBOA benefits patients with hemodynamically unstable pelvic fractures or if pelvic packing, external fixation, and/or angioembolization should be conducted without delay for REBOA application [5]. There is also a lack of evidence on if pre-hospital REBOA application should be utilized for pelvic fractures. An investigation of combat injuries showed that 18% of causalities may have benefitted from REBOA but that 83% died before reaching the hospital, leaving Smith et al. to develop a pre-hospital REBOA prototype that self-guides to placement; however experimental data on civilian pre-hospital REBOA use has not been reported [18].

Despite the lack of certainty surrounding the safety and effectiveness of REBOA for pelvic fractures, its use is recommended by TQIP, WTA, and WSES [4, 10, 11]. All three hospitals following the TQIP guideline reported REBOA is used for pelvic fracture management. Surprising, a smaller proportion of participants who followed the WTA guideline used REBOA than of those following the EAST guideline, when EAST does not recommend REBOA for pelvic fractures [8]. It appears that 43% (9/21) of participants are not adhering to the published guideline they are following regarding REBOA use.

Although there was a higher proportion of high-volume trauma centers who used REBOA than at low-volume trauma centers, this difference was not significant. A previous study reported that high-volume trauma centers reduce all-cause patient mortality and suggest the quality of care is the underlying reason for this discrepancy, as the higher volume centers often use best-practice strategies, have a higher nurse-to-patient ratios, use multidisciplinary teams, and have targeted therapeutic treatment strategies [19].

It is no surprise that hemodynamic instability was reported as a reason for using REBOA, as the primary use for REBOA is as an adjunct to definitive hemorrhage control for patients with non-compressible torso hemorrhage and is not indicated solely for pelvic trauma [7]. The ACS states that REBOA is for patients in hemorrhagic shock who are unresponsive or transiently responsive to resuscitation for hemorrhage below the diaphragm and additionally suggest Zone 3 deployment for patients with severe pelvic hemorrhage [7]. Additional indications for REBOA use in this survey were: negative FAST, ineligible for angioembolization, and IR unavailability. The latter two reasons, ineligibility for angioembolization or unavailability of IR, have not been outlined specifically as reasons to deploy REBOA.

There were limitations to this study. The sample size was small including 40 participants, however that represents 25% of the ACS-verified Level I trauma centers in 2018. We did not ask questions regarding the zone application for REBOA, or why REBOA is *not* used. Additionally, mortality data were not collected so we are unable to make recommendations on the use of REBOA in terms of preventing mortality.

## Conclusions

There is little consensus at Level I trauma centers regarding the use of REBOA for pelvic fracture management. Roughly half of Level I trauma centers used REBOA for pelvic fracture management. Not surprisingly, hemodynamic instability was consistently reported as an indicator for REBOA in patients with pelvic fractures. In addition to hemodynamic instability, participants reported that REBOA is used when IR is unavailable, or the patient is not a candidate for angioembolization. The absence of patterns in REBOA practices for pelvic fracture management across Level I trauma centers is indicative of both the lack of evidence supporting the use of REBOA and varying recommendations in guidelines. Further research is needed to determine the safety, effectiveness, and complications of REBOA for pelvic fracture management so that practice guidelines can be updated to make recommendations according to study results.

## Data Availability

Data for this study is stored on Sharefile, an electronic HIPAA and HITECH-compliant platform that ensures all transmissions are fully encrypted, end-to-end. The datasets used for analysis for the current study are available from the corresponding author on reasonable request.

## Appendix

### Appendix 1: Level I Trauma Centers Invited to Participate in the Survey

Albany Medical Center, Banner University Medical Center – Tucson, Banner University Medical Center Phoenix, Barnes-Jewish Hospital, Baylor University Medical Center at Dallas, Baystate Medical Center, Beaumont Hospital - Royal Oak Campus, Bellevue Hospital Center, Ben Taub Hospital - Harris Health System, Beth Israel Deaconess Medical Center, Boston Medical Center, Brigham and Women’s Hospital, Bronson Methodist Hospital, Brooke Army Medical Center, Carilion Roanoke Memorial Hospital, Carolinas Medical Center, Cedars-Sinai Medical Center, Charleston Area Medical Center, Christiana Care Health System, Cleveland Clinic Akron General, Community Regional Medical Center, Cooper University Health Care, Dartmouth-Hitchcock Medical Center, Dell Seton Medical Center at the University of Texas, Denver Health Medical Center, Detroit Receiving Hospital, Dignity Health Chandler Regional Medical Center, Dignity Health St. Joseph’s Hospital and Medical Center, Duke University Hospital, East Texas Medical Center Tyler, Erie County Medical Center, Eskenazi Health, Froedtert Hospital, George Washington University Hospital, Grady Memorial Hospital, Grant Medical Center, Greenville Memorial Hospital, Harbor UCLA Medical Center, Hartford Hospital, Hennepin County Medical Center, Henry Ford Hospital, Highland Hospital/A member of Alameda Health System, HonorHealth John C. Lincoln Medical Center, HonorHealth Scottsdale Osborn Medical Center, Howard University Hospital, Hurley Medical Center, Indiana University Health Methodist Hospital, Inova Fairfax Hospital, Intermountain Medical Center, Iowa Methodist Medical Center, Jackson Memorial Hospital, Jacobi Medical Center, Jamaica Hospital Medical Center, JPS Health Network, Kendall Regional Medical Center, LAC + USC Medical Center, Legacy Emanuel Medical Center, Lincoln Medical and Mental Health Center, Loyola University Medical Center, Maine Medical Center, Maricopa Integrated Health System - Maricopa Medical Center, Massachusetts General Hospital, Mayo Clinic Rochester Trauma Centers, Medical Center Navient Health, Medical University of South Carolina, MedStar Washington Hospital Center, Memorial Hermann Hospital System – Houston, Memorial Regional Hospital, Mercy Health - St. Elizabeth Youngstown Hospital, Mercy Health - St. Vincent Medical Center, Methodist Dallas Medical Center, MetroHealth Medical Center, Miami Valley Hospital, Morristown Medical Center, Nassau University Medical Center, Nebraska Medicine - Nebraska Medical Center, New Jersey Trauma Center at the University Hospital, New York Presbyterian Hospital - Weill Cornell Medical Center, New York-Presbyterian – Queens, North Memorial Health Hospital, Northwell Health North Shore University Hospital, Northwell Health Staten Island University Hospital, NYC Health and Hospitals – Elmhurst, NYC Health and Hospitals - Kings County, NYU Langone Hospital – Brooklyn, NYU Winthrop Hospital, Oregon Health & Science University, OU Medical Center, Palmetto Health Richland, Parkland Health & Hospital System, Penrose Hospital, ProMedica Toledo Hospital, Regions Hospital, Rhode Island Hospital, Richmond University Medical Center, Robert Wood Johnson University Hospital, Ronald Reagan UCLA Medical Center, Santa Barbara Cottage Hospital, Santa Clara Valley Medical Center, Scott & White Memorial Hospital – Temple, Scripps Mercy Hospital, Sparrow Hospital, Spectrum Health - Butterworth Hospital, SSM Health Saint Louis University Hospital, St. Anthony Hospital, St. Joseph Mercy Hospital - Ann Arbor, St. Vincent Indianapolis Hospital, Stanford Health Care, Stony Brook Medicine, Summa Akron City Hospital, Swedish Medical Center, Tampa General Hospital, Medical City Plano, The Ohio State University Wexner Medical Center, The Queen’s Medical Center, The University of Kansas Hospital, The University of Toledo Medical Center, Tufts Medical Center, UC Irvine Health, UC San Diego Medical Center, UMASS Memorial Medical Center, University Health System - San Antonio, University Health-Shreveport, University Hospitals Cleveland Medical Center, University Medical Center – Lubbock, University Medical Center New Orleans, University Medical Center of El Paso, University Medical Center of El Paso, University Medical Center of Southern Nevada, University Medical Center of Southern Nevada, University of Alabama at Birmingham Hospital, University of Arkansas for Medical Sciences, University of California, Davis Medical Center, University of Cincinnati Medical Center, University of Iowa Hospitals & Clinics, University of Kentucky Albert B. Chandler Hospital, University of Louisville Hospital, University of Michigan Health System, University of Missouri Health System, University of New Mexico Hospital, University of North Carolina Hospital, University of Rochester Medical Center/Strong Memorial Hospital, University of Tennessee Medical Center, University of Texas Medical Branch, University of Utah Health Care, University of Vermont Medical Center, University of Virginia Health System, University of Wisconsin Hospital and Clinics Authority, Upstate University Hospital, Vanderbilt University Medical Center, Via Christi Hospitals – Wichita, Vidant Medical Center, Virginia Commonwealth University Medical Center, Wake Forest Baptist Medical Center, WakeMed Health & Hospitals, Wesley Medical Center**,** West Virginia University Hospitals-J.W. Ruby Memorial Hospital, Westchester Medical Center, Yale-New Haven Hospital, and Zuckerberg San Francisco General Hospital and Trauma Center.

## Abbreviations

ACS: American College of Surgeons
ATLS: Advanced Trauma Life Support
DPA: Diagnostic peritoneal aspiration
EAST: Eastern Association for the Surgery of Trauma
FAST: Focused assessment with sonography in trauma
IR: Interventional Radiology
REBOA: Resuscitative endovascular balloon occlusion of the aorta
US: United States
WSES: World Society of Emergency Surgeons
